# Trainees’ perspectives on sickle cell education: a qualitative needs assessment

**DOI:** 10.1186/s12909-024-05696-5

**Published:** 2024-07-02

**Authors:** Elizabeth J. Prince, Katherine J. Feder, Cecelia Calhoun, Alfred I. Lee, C. Patrick Carroll, Valentina Restrepo, Layla Van Doren

**Affiliations:** 1 Department of Psychiatry and Behavioral Sciences, Johns Hopkins University School of Medicine, 600 N. Wolfe St. - Meyer 112, Baltimore, MD 21287 USA; 2 Department of Medicine, Yale School of Medicine, 333 Cedar Street - Room WW201, New Haven, 06520 CT USA; 3 Section of Hematology, Yale School of Medicine, New Haven, CT, USA

**Keywords:** Sickle cell disease, Medical education, Hematology & oncology fellowship, American College of Graduate Medical Education (ACGME)

## Abstract

**Background:**

Sickle cell disease (SCD) exemplifies many of the social, racial, and healthcare equity issues in the United States. Despite its high morbidity, mortality, and cost of care, SCD has not been prioritized in research and clinical teaching, resulting in under-trained clinicians and a poor evidence base for managing complications of the disease. This study aimed to perform a needs assessment, examining the perspectives of medical trainees pursuing hematology/oncology subspecialty training regarding SCD-focused education and clinical care.

**Method:**

Inductive, iterative thematic analysis was used to explore qualitative interviews of subspecialty hematology-oncology trainees’ attitudes and preferences for education on the management of patients with SCD. Fifteen trainees from six programs in the United States participated in 4 focus groups between April and May 2023.

**Results:**

Thematic analysis resulted in 3 themes: 1. Discomfort caring for patients with SCD. 2. Challenges managing complications of SCD, and 3. Desire for SCD specific education. Patient care challenges included the complexity of managing SCD complications, limited evidence to guide practice, and healthcare bias. Skill-building challenges included lack of longitudinal exposure, access to expert clinicians, and didactics.

**Conclusions:**

Variations in exposure, limited formal didactics, and a lack of national standardization for SCD education during training contributes to trainees' discomfort and challenges in managing SCD, which in turn, contribute to decreased interest in entering the SCD workforce. The findings underscore the need for ACGME competency amendments, dedicated SCD rotations, and standardized didactics to address the gaps in SCD education.

**Graphical Abstract:**

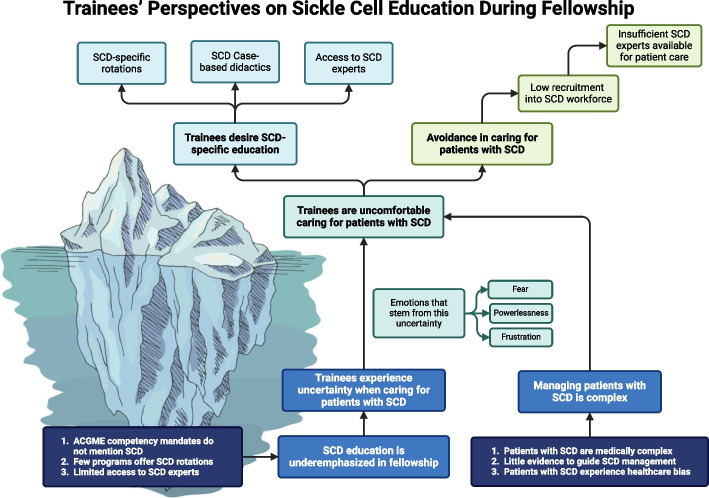

**Supplementary Information:**

The online version contains supplementary material available at 10.1186/s12909-024-05696-5.

## Introduction

Sickle cell disease (SCD) lies at the intersection of social, racial, and healthcare equity in the United States. It is the most common monogenic blood disorder worldwide, affecting at least 100,000 Americans with this number expected to increase. [1] SCD occurs in one out of 365 African American births and the trait occurs in one out of 13 African American births. [1] It is a devastating illness, with increased morbidity and mortality, and reduction in quality of life compared to persons without SCD [2–4]. Despite a high prevalence and severe complications, clinical care and research into SCD has not historically been prioritized, contributing to healthcare inequity in this population. A combination of underfunding in research, few disease modifying therapies, clinician bias, and an insufficient medical workforce with SCD expertise limits progress in improving care for persons with SCD [5].

The number of physicians trained and available to treat adults with SCD is insufficient to meet the needs of this population [6]. Clinicians describe discomfort managing patients with SCD, and have a poor understanding of SCD related complications [7, 8]. Patients and clinicians identify poor clinician knowledge about SCD as a barrier to receiving and providing quality care [9]. Clinical practice guidelines are one approach to improving clinician knowledge. The 2014 National Heart Lung and Blood Institute (NHLBI) “Evidence-Based Management of Sickle Cell Disease: Expert Panel Report,” was the first clinical practice guideline developed for SCD [10]. In 2019, the American Society of Hematology (ASH) began releasing a series of clinical practice guidelines. However, even with knowledge of existing clinical guidelines, it is often difficult for clinicians to integrate guidelines into practice, thus leading to most persons with SCD receiving non-standard care [11].

Over three decades ago, insufficient emphasis on SCD education within medical education was noted [12]. This was due to an already overcrowded curricula with SCD thought to be a rare disease in the U.S. at that time and less urgency to learn about SCD than human immunodeficiency virus (HIV). Patient-oriented one-on-one teaching encounters were thought to be most beneficial in improving knowledge of SCD. The Accreditation Council for Graduate Medical Education (ACGME) uses milestones as a framework for the assessment of fellow development in key dimensions of physician competency [13]. Hematology/oncology fellowship training requirements from the ACGME mandate competency in each specific area of oncology (i.e. breast, gynecologic, lung), while all of classical hematology—including SCD—is summed up as a single competency in "acquired and congenital disorders of red cells, white cells, and platelets.” [14] Even ACGME-accredited hematology/oncology fellowship training program websites inadequately feature classical hematology training [15]. As the majority of hematology/oncology trained physicians opt for a career in oncology, the pool of practitioners with expertise in SCD continues to diminish [16].

There is no universal curriculum for SCD education, nor research detailing the most effective ways to train clinicians to manage this complex disease. SCD has been historically under-funded and under-treated relative to other chronic conditions, and there is an urgent need to address this gap in hematology/oncology training. The management of SCD is at the precipice of new therapies, including gene therapy, and clinicians’ expertise in the evaluation of the illness before, during, and after exposure to these intensive therapies is urgently needed. This study is the first to examine perspectives of medical trainees pursing subspecialty training in hematology and oncology with regards to SCD focused education and clinical care.

## Methods

### Recruitment and Sampling

This study was determined to be exempt under Yale University institutional review board in February 2023 and all participants provided written informed consent prior to focus group participation. Seven hematology-oncology training program directors were emailed (by author LV) between February and March 2023 to request trainee contact information for focus group invitation. All participating institutions were in urban centers of the United States with a high prevalence of SCD. Institutions with and without a sickle cell program were selected to comprehensively evaluate the sickle cell specific educational needs at institutions with different sickle cell disease care models.

Current hematology/oncology fellows and senior residents applying for hematology/oncology fellowship were recruited. A convenience sampling approach was used based on trainee willingness and availability to participate in focus groups. Twenty-four trainees were invited via email by LV to participate in a focus group. Prior to focus group discussions, demographic information was collected on awareness and educational utility of the NHLBI guidelines, ASH guidelines, and ASH education videos for SCD. Responses to the demographic questionnaire informed the development of the focus group guide. 15 trainees participated in the focus groups (Table 1). Focus group participants at four of the six institutions had a sickle cell disease program. Some institutions allowed for a dedicated SCD rotation or required fellows to directly care for persons with SCD on a primary hematology service or hematology consult service. Other programs did not have rotation on a sickle cell service. Participants received a gift card for focus group participation.

**Table 1:** Focus group participant trainee level and training institution characteristics. g. Trainees from institution G responded to the survey, but none participated in the focus group

	*N *= 15	%
**Hematology fellow**	13	90
Adult	10	67
Pediatric	3	20
**Internal medicine resident**	2	10
**Training institution**		
Site A	5	33
Site B	3	20
Site C	3	20
Site D	2	13
Site E	1	7
Site F	1	7
Site G^g^	0	0
Sickle Cell Disease Program	10	67
Medium (7–21 fellows)	10	67
Large (> 21 fellows)	5	33

### Data collection

A semi-structured focus group guide was developed by authors LV, CC, and CPC based on the results of the pre-focus group questionnaire and professional experience in medical education, inquiring about trainee preferences and attitudes for education on the management of patients with SCD (Supplement 1). We began the focus group by asking participants to reflect on a time they ran across a medical problem with a patient with SCD and did not know how to manage the complication. Four 50-to-60-min focus group sessions with four to five participants each were completed between April 2023 and May 2023 via zoom. Reflective clarifying questions invited participants to expand on their responses. Focus groups were conducted by LV, who had worked clinically with five of the participants of the focus groups. All focus groups were recorded and transcribed using Otter.ai and reviewed by LV to ensure transcription accuracy.

### Analysis

Focus group transcripts were double coded by an academic hematologist specializing in SCD (LV) and an academic psychiatrist specializing in SCD (EP) using qualitative analysis software NVivo (Version 12). We used an inductive and iterative thematic analysis coding approach as described by Braun & Clarke [18]. Transcripts were analyzed line by line, segmented into meaningful analytical units, and marked with descriptive words (coding), and a list of codes (code list) was generated and reapplied to new segments of data. Once coding was completed by both coders for all transcripts, the contents were compared and discussed to achieve consensus regarding themes and subthemes. The final number of focus group participants was sufficient to provide rich data that allowed for robust themes. Analysis and reporting of our qualitative research followed the Standards for Reporting Qualitative Research (SRQR) [19].

## Results

### Pre-focus group demographic questionnaire

All focus group participants completed the pre-focus group demographic questionnaire to inform the focus group guide (Table 2). This ensured a mix of perspectives from those who were aware of the guidelines compared to those who were not. Eighty- percent (12/15) were aware of NHLBI and/or ASH SCD guidelines. Of those who were aware of the guidelines, 58% (7/15) found them helpful in managing patients with SCD, while the remainder were neutral or disagreed that they were helpful. Only 40% (6/15) of trainees were aware of the ASH SCD education videos, with 50% (3/6) finding them helpful. Focus group participants expressed the concern with the applicability of the NHLBI and ASH SCD guidelines into clinical practice given the low quality of evidence that forms the basis for most guidelines.

**Table 2:** Sickle cell guideline survey responses

Questionnaire respondents	N = 15	%
**Aware of NHLBI or ASH guidelines**	12	80
Found guidelines helpful	7	58
**Aware of ASH SCD education videos**	6	40
Found videos helpful	3	50

*NHLBI* National Heart, Lung, and Blood Institute, *ASH* American Society of Hematology, *SCD* Sickle Cell Disease

### Focus group themes

Thematic analysis of trainees’ attitudes and preferences for education on the management of patients with SCD identified three major themes (Table 3): 1. Discomfort caring for patients with SCD. 2. Challenges managing complications of SCD, and 3. Desire for SCD specific education. Each theme is described below and supported with key quotes from participants.

**Table 3:** Trainee perspectives on the challenges and components of sickle cell disease education: themes and subthemes exemplar quotes

Themes	Subthemes and quotes		
Trainees experience discomfort	**Fear** “I’m afraid because I don’t know if I’m going to know what to do.”	**Frustration** “The frustration that I feel… comes from a sensation that I don’t have something to offer.”	**Powerlessness** “Feeling in some ways powerless to take away some of their pain… and feeling a little bit intimidated.”
Trainees find SCD management challenging	**Medical Complexity** “The physiology is complicated… they have a lot of things going on.”	**Limited Evidence** “There’s so much variation in practice… because there’s such a lack of evidence.”	**Healthcare Bias** “There’s a lot more stigma associated with sickle cell disease… patients have to put up with a lot more negative aspects of our healthcare system.”
Trainees desire SCD specific education	**Exposure to Patients** “More exposure would definitely have made me more comfortable.”	**Access to Expertise** “The most important thing for me is having access to providers where this is their area of expertise.”	**Didactics** “Didactics are especially helpful in this area, where there’s so much ambiguity.”

**1. Discomfort caring for patients with SCD** – "The constant uncertainty makes it more uncomfortable."

Many trainees shared feeling uncomfortable caring for patients with SCD due to uncertainty and lack of confidence that they can ameliorate the patients’ suffering.

“You’re always kind of operating in a gray area, and you never really feel like you have a good handle about what’s going on. It’s rare that you feel 100% certain. The constant uncertainty makes it more uncomfortable."

Multiple participants experienced fear when caring for patients with SCD.

“It’s the middle of the night and I get a page, it’s a sickle cell patient from the ER. I’m afraid because I don’t know if I’m going to know what to do. It’s that uncomfortableness of ‘I don’t know if I can do this by myself.’ Fellowship has given me an appreciation for how severe sickle cell disease can be and all the complications that can go down very quickly.”

Negative emotions experienced by trainees may mirror the emotions of the patients themselves: frustration, powerlessness, and even helplessness.

“There was a lot of negative feelings associated with sickle cell patients. I think part of that comes from our inability to truly understand their pain, how to manage pain in sickle cell disease.”

“Patients are admitted [repeatedly] for the same symptoms, it’s so sad to see. We really don’t know how to help them in meaningful ways. That’s when I feel more helpless when it comes to caring for these patients.”

Participants described how discomfort related to pain treatment could negatively impact provider attitudes.

“Because a lot of people are not familiar and they’re not comfortable [managing pain] they put it on the patient as ‘drug seeking behavior.' It’s unfortunate how we don’t truly understand, and patients get mis-labeled.”

However, discomfort around management of pain was reduced by individualized treatment plans.

“If it weren’t for pain plans, I would feel uncomfortable. If those weren’t there, I would have a harder time figuring out what’s going to be best for this patient.”

Discomfort with SCD was different from other hematologic emergencies, and participants explained how they became more comfortable with some conditions during training, but the discomfort with SCD persisted.

“At the beginning of the year, every call was scary. But now, if someone has AML or TTP, I have a good sense of what to do. At the end of first year of fellowship, the major thing I’m afraid of is SCD.”

**2. Challenges caring for patients with SCD**—“They have a lot of things going on.”

Caring for patients with SCD was experienced as more difficult compared to caring for patients with other chronic illnesses (i.e. CHF, cystic fibrosis). This sentiment was driven by the paucity of high-level evidence to guide management, variation in practice amongst experts, and the lack of objective measurement tools for the most common presentation, acute pain.

“Sickle cell is more complicated than acute leukemia or TTP, in terms of management, even after you get exposed to all of them, because sickle cell can present in a more variety of ways. There are a lot more decision-making points and there are many more things to think about."

"There's so much variation in practice because there's a lack of evidence."

“There’s a lot less research and data behind treatments for sickle cell compared to something like [congestive heart failure].”

Participants familiar with the ASH and NHLBI SCD guidelines discussed their value and limitations for management of patients.

“In some instances where there is more evidence, the guidelines are clearer, but I feel like there’s certain areas where there is not as much evidence, and [the guidelines] read kind of like expert opinions."

“[The guidelines are] a useful reference primarily for screening and what I should be keeping track of, but not as helpful on an individual ‘How do I manage this acute situation.’”

Lastly, trainees expressed that navigating bias in healthcare contributes to the challenge of caring for patients with SCD.

“So much more of caring for patients is dealing with [bias in the] healthcare system, more than with other diseases.”

“I’ve learned so much from my sickle cell patients. They have dealt with the medical system in such an extreme way that so many of our other patients haven’t. Oftentimes their whole life, and oftentimes they experience it as a marginalized person.”

## Trainees desire SCD specific education—“A lesson in life”

Most trainees reported that SCD specific education was not emphasized during their training, even those at institutions with dedicated sickle cell programs. Those where it was emphasized felt it was a valuable part of their training.

“My rotation in sickle cell was a lesson in life. I learned so much from my patients, who they are, what they’ve experienced in the healthcare system. Every single patient with sickle cell that I'd meet, I’d feel like I have learned something more about what we need to be doing better with the system.”

Not all participants felt they had adequate exposure to patients with SCD to feel equipped to care for them.

“I didn’t get as much [experience seeing patients with SCD] as I think I would need in order to feel comfortable as an attending.”

Rotations as part of specialized SCD teams were valued, but participants also wanted longitudinal care experiences.

“Learning within the interdisciplinary team has been so helpful, because that made me feel like, ‘oh wow, this is what caring for patients with sickle cell is supposed to look like,' and seeing how crucial the expertise from all the members... it was very eye opening.”

“SCD is one of the diseases that is most different on paper than in person. The most useful experience as a fellow has been immersing [myself] in sickle cell.”

Participants conveyed that access to clinical experts is a necessary component of medical education.

“You have some negative exposures, and unless you [work with] a true expert in the field, your experience is very different.”

“[SCD is] a siloed spectrum of diseases. I don’t learn nearly as much from a hematology consult attending as I do from a dedicated sickle cell person.”

Available literature and guidelines were not felt to be a replacement for experts.

“There [are] nuances that might guide a decision more than just following an algorithm on paper. The expert’s opinion matters more.”

“Even if you do the research, ultimately you’re going to end up discussing with the sickle cell attending.”

Participants recognized that SCD experts are not universally available at all institutions and suggested other approaches to access clinical experts.

“We’re very lucky to have access to incredible experts. There are tons of fellows at institutions that do not have access to sickle cell experts. I think in those settings the didactics become a lot more crucial, potentially from visiting experts."

"Remote tumor boards might be one way to provide sickle cell expertise in areas that might not have it.”

Sickle cell focused didactics varied widely between training programs. Some participants had received one or no lectures on SCD throughout training. Those with more intensive sickle cell specific teaching, including a SCD rotation, described how this made them more confident managing complications of SCD.

"I can’t think of a place, either in residency or fellowship, where we've talked formally about sickle cell and management of complications.”

Case-based learning was mentioned as a valuable strategy, especially in fellowship training.

“[Case based learning] helps you think about [the patient] versus thinking about some abstract complication.”

**Fig. 1:**
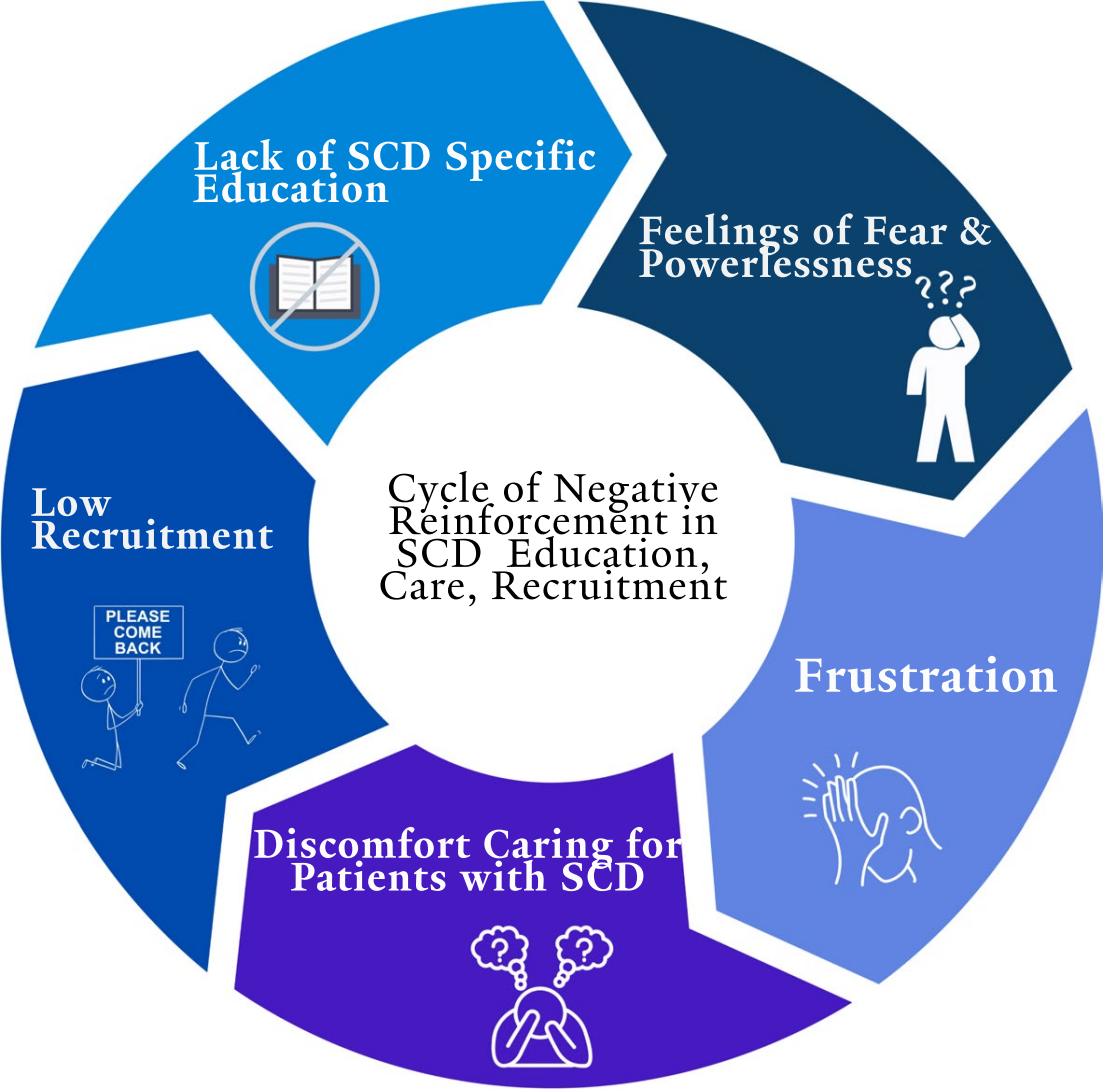
Cycle of negative reinforcement in SCD education, care, and recruitment

Overall, participants connected a lack of specific education on caring for people with SCD to a cycle of discomfort and avoidance, and fewer clinicians’ desire to specifically treat them (Fig. 1).

"Most people like doing things they are better at, and they don’t want to harm anyone. ‘Am I going to be the best doctor for this patient?’ I don’t want to do the wrong thing. It makes it less comfortable and then it becomes less desirable."

“If people don’t see patients with sickle cell that often it may not be reasonable to expect them to get to the point where they're truly comfortable. But we also don’t want to say patients can only go to certain centers, because then you’re going to have issues with access to care. How do you meet in the middle there?”

## Discussion

In this study we find high concern about the state of SCD education in the US. Our study is the first to examine trainee perspectives on SCD specific education and clinical care during hematology/oncology fellowship training. Overall, SCD specific education is under-emphasized in hematology/oncology fellowship, even at institutions with a high prevalence of SCD and a sickle cell program. New treatments for SCD and promoting healthcare equity heighten the need for a competent workforce of subspecialty trained physicians able to care for persons with SCD.

Participants cited inadequate clinical exposure, insufficient access to SCD expertise, and a lack of curricula for sickle cell education. The themes were consistent despite heterogeneity in training programs’ exposure to patients and access to clinical experts. Focus group participants at four of the six institutions had a sickle cell program. The variation in exposure to patients and formal didactics among programs reflects the lack of national standardization in training for SCD. This demonstrates that the presence of more comprehensive resources for SCD at certain institutions does not automatically translate to prioritizing SCD education for trainees, such as through dedicated SCD rotations.

Fellows describe feeling uncomfortable managing SCD, but none expressed negative attitudes towards this population. Discomfort is an appropriate response to managing a medically complex illness with little data to guide management and few treatment options. However, discomfort and frustration have the potential to develop into avoidance and even negative attitudes. Negative provider attitudes and bias in SCD jeopardize delivery of quality care, but educational interventions improve attitudes and care delivery [20, 21]. Participants in this study recognized a lack of understanding can lead to negative provider attitudes, and access to clinical experts in this area attenuates negative exposures during training. Participants also expressed that navigating bias within the healthcare system added a level of stress in caring for patients with SCD. Ensuring a comprehensive SCD training experience during hematology/oncology fellowship is therefore vital in reducing healthcare bias.

Most participants were aware of the ASH and NHLBI clinical practice guidelines for SCD and found them helpful for some situations, such as stroke screening or management of acute stroke where the data is more robust. However, they also acknowledged their limitations for management of most clinical challenges of SCD where individualized care is required. Despite being highly motivated to engage in self-directed learning, some trainees reported feeling discouraged by the absence of a clinical expert at their institution. The absence of a sickle cell expert at the institution impeded their learning, as the majority of guideline-based practice recommendations are conditional recommendations with very low certainty in the evidence about the effects [22, 23]. Certain guidelines, such as the ASH SCD guideline for managing acute and chronic pain, offer recommendations that assume the presence of a SCD clinical expert within the institution where one might not be available [24]. To better incorporate patient care into standardized guidelines, specific institutional protocols can be developed centered around the ASH/NHLBI guidelines detailing management strategies for common acute complications of SCD, such as acute chest syndrome, pain crises, and stroke. Exposure to patients, access to SCD experts, and sickle cell specific didactic curricula were three domains identified by participants as necessary components of SCD specific education during training.

Participants expressed that sickle cell specific education would make them more comfortable managing SCD. Disparities in access to SCD experts or educational resources across institutions can be a barrier to education. Remote learning didactics or a “virtual tumor board” were suggestions to improve access to clinical experts at institutions where an expert might not otherwise be available. For example, existing ECHO tele-mentoring programs are one approach to improving access to SCD experts for case-based learning and didactics [25]. Collaborative networks, such as ASH SCD Centers Workshop, are another source of education since establishing partnerships with larger, comprehensive SCD centers or SCD networks can facilitate knowledge sharing and provide trainees with opportunities for short-term rotations. Virtual consulting platforms for non-urgent questions, such as themednet.org or ASH Consult a Colleague are also potential opportunities for learning. While structured didactics are helpful in building foundational knowledge, experiential learning cultivates comfort and practice-specific expertise [26]. In our study, trainees described variations in clinical management between clinical experts as contributing to the uncertainty in management. While this might be true in our current study, observing clinical practice variability in the form of individualized patient care can be useful for the learner in certain disease states [27]. Trainees who felt the most comfortable received more exposure to persons with SCD in fellowship, in the form of a primary hematology service where patients with SCD were admitted, or a dedicated sickle cell rotation with an inpatient and outpatient component. A hematology-general medicine hybrid team was recently shown to improve knowledge in the management of SCD and hematology attendings reported increased opportunities for teaching [28].

Overall, participants outlined a pattern where inadequate training, resulting in discomfort managing patients with SCD, leads to a reduced desire to enter the SCD workforce. The shortage of specialists in the care of persons with SCD, especially in adult care, hinders access to comprehensive care [6]. Prioritizing sickle cell education during fellowship could potentially mitigate feelings of discomfort and increase interest in entering the SCD workforce. We also acknowledge that trainees’ uncertainty could result from not having gained maturity as clinicians; however, even at the end of the first year of fellowship, one trainee acknowledged comfort managing more rare hematologic diseases, such as acquired thrombocytopenic thrombotic purpura and acute myeloid leukemia.

This study has limitations. Institutions were selected in areas with a high prevalence of persons living with SCD, limiting perspectives of trainees in areas with a lower prevalence of SCD. The convenience sampling approach limited the perspectives to trainees willing and available to participate in focus groups. Participant demographic information, such as gender and race were not obtained. Prior studies have shown an association of provider race and attitude in caring for persons with SCD [29]. Although the focus group moderator (LV) had worked clinically with some of the focus group participants, the semi-structured focus group guide was consistently applied across groups.

It is also worth noting that only three participants were pediatric hematology/oncology fellows. Although the themes were consistent, this small sample size does make it difficult to generalize the results of this study to pediatric training programs. Historically, SCD was considered an illness of childhood with most not living until adulthood. This has led to a higher concentration of expertise among pediatric providers compared to adult providers, directly impacting the training of future healthcare professionals. Although the most robust data on managing SCD complications, namely stroke, comes from pediatric literature, the overall evidence base for SCD management remains inadequate. Overall, there remains a global shortage of both pediatric and adult providers qualified and willing to care for persons with SCD, thus illustrating the need for improvement in educational curriculum broadly. A follow-up study with majority pediatric-focused participants would be valuable to explore how themes differ between adult and pediatric training programs.

## Conclusion

An amendment to ACGME competency mandates is required to include proficiency in managing patients with SCD. We encourage hematology/oncology fellowship programs to: 1. acknowledge the discomfort/lack of confidence experienced by trainees to foster a supportive learning climate; 2. Integrate dedicated SCD rotations into fellowship training; 3. Develop classroom and case-based didactics to reinforce clinical principles. Finally, conducting a nationwide needs assessment is essential to further define the landscape of SCD education in fellowship training across various SCD care models to select the best conceptual framework for educational intervention design.

### Supplementary Information

Supplementary Material 1.

## Data Availability

The datasets used and/or analyzed during the current study are available from the corresponding author on reasonable request.
