# Supplementary material for: Trainees’ perspectives on sickle cell education: a qualitative needs assessment
Source: BMC Med Educ. 2024 Jul 2;24:715. doi: 10.1186/s12909-024-05696-5 (PMC11220977; doi:10.1186/s12909-024-05696-5)
Supplement: Supplementary file 1 — Supplementary Material 1. [file 12909_2024_5696_MOESM1_ESM.pdf]

Interview Questions (Semi-structured)

- How does caring for patients with SCD differ from caring for other patients with a chronic illness?
  - Probe: What is it about caring for patients with SCD is difficult or uncomfortable?
  - Probe: How do you approach these differences?
- When was the last time you ran across a problem with a patient with SCD that you did not know how to handle yourself?
  - Probe: What made you uncomfortable or unprepared about taking care of the patient?
  - Probe: How did you navigate this interaction?
- What previous education have you had on the management of sickle cell disease?
  - Probe: Did you feel prepared to care for patients with SCD after the education session?
  - Probe: what did you find effective or not effective about that educational session?
- Would receiving education for managing patients with SCD make you more comfortable taking care of this population?
  - Probe: what educational activity would be most effective (simulation, lecture, on the job)?
- Does the gap in education make it less desirable to care for patients with SCD?
- What do you think are the unique needs for *hospitalists* receiving education regarding the management of sickle cell disease?
- Is there anything else about how to best education and empower providers to care for persons with SCD that you would like to share?
